# The effect of interventional program underpinned by health belief model on awareness, attitude, and performance of nurses in preventing nosocomial infections: A randomized controlled trial study

**DOI:** 10.17533/udea.iee.v41n3e10

**Published:** 2023-10-26

**Authors:** Mahmoud Hosseinpour, Rasool Eslami Akbar, Mohsen Faseleh Jahromi, Zohreh Badiyepeymaiejahromi

**Affiliations:** 1 Nurse, MSc. Student Research Committee. Email: m.hossinpour98arshad@gmail.com m.hossinpour98arshad@gmail.com; 3 Nurse, Ph.D. Assistant Professor. School of Nursing. Email: mohsenefaseleh@yahoo.com School of Nursing mohsenefaseleh@yahoo.com; 4 Nurse, Ph.D. Assistant Professor. Email: z.badiyepeyma@gmail.com z.badiyepeyma@gmail.com; 5 Jahrom University of Medical Sciences, Jahrom, Iran. Jahrom University of Medical Sciences Jahrom Iran

**Keywords:** Health belief model, Cross infection, nurses, attitude of health personnel, control groups, randomized controlled trial, modelo de creencias sobre la salud, infección hospitalaria, enfermeras y enfermeros, actitud del personal de salud, grupos controles, ensayo clínico controlado aleatorio, modelo de crenças de saúde, infecção hospitalar, enfermeiras e enfermeiros, atitude do pessoal de saúde, grupos controle, ensaio clínico controlado aleatório

## Abstract

**Objective.:**

The present study examined the effect of an interventional program underpinned by the Health Belief Model (HBM) on nurses’ awareness, attitude, and performance in preventing nosocomial infections.

**Methods.:**

This randomized controlled trial study was performed on 60 clinical nurses in lar, Iran. Nurses were selected using the simple random sampling method and assigned to two experimental (*n*=30) and control (*n*=30) groups. Data collection tool included the valid and reliable questionnaire was developed by Soleimani *et al.* The research intervention consisted of five 90-min sessions based on the health belief model in preventing hospital infection for experimental group. Before the intervention, immediately and two months after the intervention, the two groups completed the questionnaire. The control group received no intervention.

**Results.:**

Data analysis showed that the differences between the two groups was statistically significant immediately and two months after the intervention (*p*<0.05). In experimental group the changes in the mean score of knowledge, attitude and performance of nurses before, immediately and two months after the intervention were significant (*p*<0.05), but in the control group, only the changes in the mean score of performance were significant (*p*<0.05).

**Conclusion.:**

The results showed that the HBM-based intervention is effective in promoting nurses’ knowledge, attitude, and performance in preventing nosocomial infections. hence, periodical and in-service HBM-based training programs on preventing nosocomial infections are recommended to be held for nurses.

## Resumen

**Objetivo.:**

El presente estudio examinó el efecto de un programa de intervención basado en el modelo de creencias en salud (Health Belief Model -HBM-, en inglés sobre el conocimiento, la actitud y el desempeño de las enfermeras en la prevención de las infecciones intrahospitalarias.

**Métodos.:**

Este ensayo controlado aleatorizado se realizó en 60 enfermeras clínicas de lar, Irán. Las enfermeras fueron seleccionadas mediante el método de muestreo aleatorio simple y asignadas a dos grupos experimental (*n*=30) y de control (*n*=30). La herramienta para la recogida de datos incluyó el cuestionario válido y fiable desarrollado por Soleimani *et al.* La intervención consistió en cinco sesiones de 90 minutos basadas en el modelo de creencias de salud para prevenir la infección intrahospitalaria en el grupo experimental. Antes de la intervención, inmediatamente y dos meses después de la intervención, los dos grupos completaron el cuestionario. El grupo de control no recibió ninguna intervención.

**Resultados.:**

El análisis de los datos mostró diferencias estadísticamente significativas entre los dos grupos en los momentos inmediatamente y dos meses después de la intervención (*p*<0.05). En el grupo experimental, los cambios en la puntuación media de conocimientos, actitudes y rendimiento de las enfermeras se observaron en los momentos de antes, inmediatamente y dos meses después de la intervención (*p*<0.05); mientras que en el grupo de control solamente los cambios en la puntuación media de desempeño fueron significativos (*p*<0.05).

**Conclusión.:**

Los resultados mostraron que la intervención basada en HBM fue eficaz para promover el conocimiento, la actitud y el rendimiento de las enfermeras en la prevención de las infecciones intrahospitalaria, por lo que se recomienda impartir a las enfermeras programas de formación periódicos y en servicio basados en HBM sobre la prevención de las infecciones intrahospitalarias.

## Resumo

**Objetivo.:**

Examinar o efeito de um programa de intervenção baseado no Modelo de Crenças em Saúde (MBH) no conhecimento, atitude e desempenho dos enfermeiros na prevenção de infecções hospitalares.

**Métodos.:**

Este ensaio clínico randomizado foi conduzido em 60 enfermeiras clínicas de lar, Irã. Os enfermeiros foram selecionados pelo método de amostragem aleatória simples e distribuídos em dois grupos experimental (*n*=30) e controle (*n*=30). O instrumento de coleta de dados incluiu o questionário válido e confiável desenvolvido por Soleimani et al. A intervenção consistiu em cinco sessões de 90 minutos baseadas no modelo de crenças em saúde para prevenir infecção hospitalar no grupo experimental. Antes, imediatamente e dois meses após a intervenção, ambos os grupos responderam ao questionário. O grupo controle não recebeu nenhuma intervenção.

**Resultados.:**

A análise dos dados mostrou diferenças estatisticamente significativas entre os dois grupos imediatamente e dois meses após a intervenção (*p*<0.05). No grupo experimental foram observadas alterações na pontuação média de conhecimentos, atitudes e desempenho dos enfermeiros antes, imediatamente e dois meses após a intervenção (*p*<0.05); enquanto no grupo controle apenas as alterações na pontuação média de desempenho foram significativas (*p*<0.05).

**Conclusão.:**

Os resultados demonstraram que a intervenção baseada no HBM foi eficaz na promoção do conhecimento, atitude e desempenho dos enfermeiros na prevenção de infecções hospitalares, pelo que se recomenda proporcionar aos enfermeiros este tipo de programas de formação em serviço baseados no HBM.

## Introduction

Nosocomial infections (NI) are the most common health complication threatening patients’ safety in the health system in all countries, regardless of the level of their development. NI result in deaths, delays in the recovery process, and disability and impose exorbitant costs.^1^ The World Health Organization (WHO) defines NI (hospital-acquired infections) as an infection acquired by a patient during the process of care in a hospital or other health and treatment centers which is not present or incubating at admission.^2^ NI result in permanent complications, prolonged hospital stays, severe increase in treatment costs, the patient’s and his/her companions’ dissatisfaction, and even death.^3^ They are also associated with increased antibiotic use, respiratory problems, enhanced demand for mechanical ventilation, and increased use of intravenous injections, thereby arousing patients’ dissatisfaction.^4^ According to the WHO, 1.7 million NI occur annually, and 1 out of 20 persons gets a hospital-acquired infection, accounting for 99,000 deaths per year and imposing about 26-32 million dollars costs on countries.^5^ In the United States, NI account for 80,000 deaths annually,^6^ implying that 247 persons die from NI in this country, and one out of 136 hospitalized patients becomes terribly sick because of such infections.^7^ In developing countries, however, 2-4 million NI occur per year, and they are considered the eleventh cause of death and the fifth cause of hospital death.^8^ In Iran, the prevalence of NI ranges from 1.9% to >25%.^9^

Nurses play the most critical role in preventing NI since they mainly contribute to the treatment and care of such patients. ^10^ According to the WHO’s practical guide 2002, nurses play a leadership role in NI.^11^ Previous studies have documented that nurses’ awareness, attitude, and performance play a vital role in hospitals and healthcare centers in terms of NI prevalence and control; hence, disregarding these factors would be associated with adverse consequences and cause physical and financial damage to patients and their companions, nurses, and other hospitals. In other words, nurses should acquire authentic and sufficient scientific information about NI and their prevention methods.^12^^,^^13^ From the WHO perspective, the most important principle in prevention is education.^14^ In this regard, the first and the most important method to decrease the incidence of NI is to teach how they emerge and how to prevent and fight them.^15^ Yaghoubi *et al.* ^(^^13^ reported that 87.7% of nurses had superficial knowledge about NI control and those nurses hold negative attitudes toward such infections. In their study, 78% of the nurses had an average performance in terms of NI control. In Nasiri *et al*.'s study, the mean score of the nurses’ attitude and performance in NI prevention was low; however, the nurses had enough knowledge about such infections.^16^

Hosseini *et al*.^17^ showed the effect of practical and theoretical training on the nurses’ knowledge and performance in preventing NI. In their study, however, the mean score of the nurses’ attitude did not change, suggesting that training did not cover their attitude, as a result of which the performance did not improve remarkably. In India, Chakcar *et al.*^18^ documented the positive effect of training on the nurses’ awareness and knowledge about NI prevention, even though no progress was noticed in their performance. These findings imply that training and information transfer are not enough to change nurses’ behavior and that promoting their awareness does not necessarily change their attitudes, and changing attitudes is not always associated with changes in behavior. Accordingly, health educators should develop interventions that lead to skill development, concept acquisition, and decision-making.^19^ In educational planning, the first step and one of the main measures is to choose a model or theory tailored to conditions and to recognize the problem as well as the goal of the concerned intervention.^20^ In other words, selecting an appropriate training model makes the program be initiated and move in the right direction.^21^

The health belief model (HBM) is one of the main models in health education^22^ , which is based on motivating individuals and changing their behavior and focuses on changing beliefs, ultimately leading to behavior changes.^23^ According to HBM and its key components (namely perceived susceptibility, perceived seriousness, perceived barriers, perceived benefits, self-efficacy, and guidelines for action), nursing staff are assumed to exhibit an appropriate reaction to NI when they feel themselves at risk of exposure to pathogenic agents (perceived susceptibility). In this case, they consider the risk of exposure to be of great importance for their health and patients (perceived seriousness).^24^ It is also likely that understanding benefits and knowing how to remove barriers are effective in taking standard infection control precautions nurses as such nurses can reach the required efficiency in preventing NI by promoting their awareness and attitudes.^24^ Accordingly, the multifaceted HBM constructs seem to match and overlap with NI prevention. In a similar vein, holding an interventional program underpinned by this model can play a critical role in improving nurses’ awareness and attitudes and enhancing their NI preventive behaviors. In this regard, the present study aimed to examine the effect of an HBM-based intervention on nurses’ awareness, attitudes, and performance in preventing NI. If the intervention is effective, it can be considered by health managers and researchers as an effective program in preventing and reducing NI. 

## Methods

In this randomized controlled trial study, the intervention was implementing an educational HBM-based program. This study was conducted in 2022 for three months at the Imam Reza Hospital in Larestan. The statistical population encompassed 130 nurses employed in this hospital. The sample size in each group regarding the error type I of 0.05, the effect size of 0.784, the power of 90%, and 10% attrition was 30 persons.^25^ In total, 60 nurses were selected using the simple random sampling method and assigned to two experimental and control groups. Inclusion criteria were holding at least a bachelor's degree in nursing, willingness to participate in this research, and employment in the concerned hospital as a clinical nurse. Exclusion criteria were also as follows: unwillingness to participate or continue the study, incomplete questionnaires, and being absent for more than two training sessions. The dropouts were five persons in the experimental group (because of being absent in training sessions and incomplete questionnaires; *n*=25) and four persons in the control group (because of incomplete questionnaires and pregnancy weakness; *n*=26). 

The required data was collected using a two-section questionnaire. In the first section, the participants’ demographic information, including age, gender, level of education, marital status, hospital department, work experience, and type of employment, was collected. The second section was to assess the nursing staff’s awareness, attitudes, and performance in preventing NIs. This 45-item questionnaire was developed by Soleimani *et al.*^26^ to address awareness (15 items), attitude (15 items), and performance (15 items). The clinical nurses’ awareness was determined based on the number of correct answers to the questions as such, there was 1 point for correct answers and 0 point for each wrong answer. The participants’ awareness scores ranged from 0 to 15. The participants’ attitude was scored on a 3-point Likert scale: "I agree = 3", "I have no idea = 2", and "I disagree = 1". In this regard, the minimum and maximum scores were 15 and 45, respectively. The nurses’ performance in this questionnaire was scored by some propositions addressing the degree of compliance with the NI prevention principles and rules on a 5-point Likert scale (5= always, 4= often, 3= sometimes, 2= rarely, and 1= never). The minimum score of performance was 15, and the maximum score was 75.

The validity and reliability of the concerned questionnaire measuring nurses’ awareness, attitude, and performance in preventing NI were examined by Soleimani et al. To check its content validity, they used the content validity index (CVI) and content validity ratio (CVR), both of which were in the range of 0.8-1. The test-retest method was used to measure the external reliability of the questionnaire, and the result was 0.75, reflecting the acceptable reliability of the questionnaire.^26^ Prior to the intervention, the two groups completed the questionnaire as a pre-test. Then an educational HBM-based intervention was presented in the conference hall of the Imam Reza Hospital for the experimental group during five 90-minute sessions once a week for five weeks. In this workshop, the content of the sessions was presented according to HBM and the most recent scientific articles, books, and pamphlets of the Center for Disease Control and Prevention of the United States of America. The first session dealt with perceived susceptibility and perceived intensity. In this session, there were lectures and group discussions (five groups of six) on the scientific definition of NI, the high prevalence of NI, and the transmission of microorganisms in hospitals. In the second and third sessions, the other two HBM constructs, including perceived obstacles and perceived benefits, were addressed, and the existing barriers to the prevention of NI and appropriate solutions to remove or control such barriers were discussed. The nurses were trained in NI control methods. In the fourth and fifth sessions, the guidelines for action and self-efficacy as two other HBM constructs were addressed, and there were some discussions on action guidelines, standard precautions, preventive behaviors against NI, nurses' abilities to reduce NI, and their constructive role in NI control. At the end of the intervention, the questionnaire was completed by the experimental and control groups once more. Two months after the intervention, the two groups completed the research questionnaires once more. The nurses in the control groups received no intervention. The collected data was then statistically analyzed with SPSS software version 21 at *p*=0.05.

Ethical considerations. The study was conducted after being approved by the Expert Council of the Faculty of Nursing, Jahrom University of Medical Sciences, receiving licenses from the Research Vice-Chancellor of the Jahrom University of Medical Sciences, and obtaining the code of ethics (Code: IR.JUMS.REC.1401.019). Moreover, the participants submitted their written informed consent prior to the study, and they were ensured of information confidentiality. 

## Results

**Figure 1:**
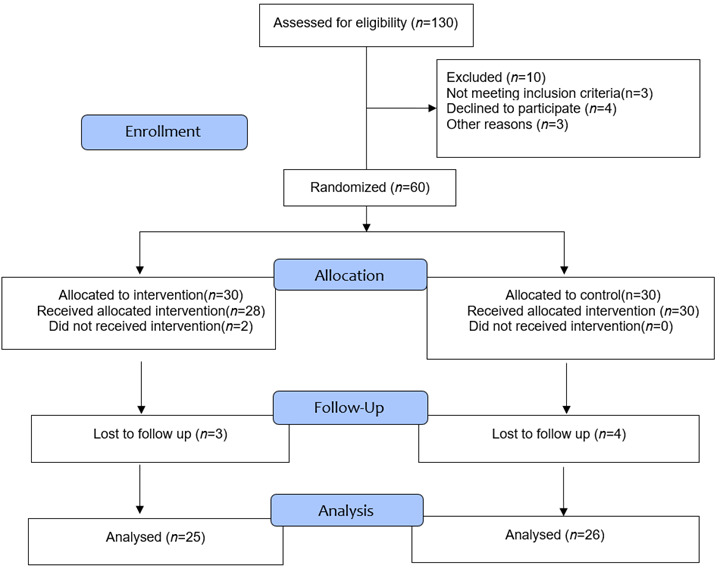
flow chart of the study

The study participants were 60 clinical nurses of the Imam Reza (AS) Hospital in Iran. (Figure 1)

Most of the nurses in the experimental (76%) and control (65.5%) groups were female. Moreover, a majority of the participants in the experimental (76%) and control (69.2%) groups were below 10 years of age. In the experimental (92%) and control (92.3%) groups, most of the nurses had <10 years of experience. Most of the nurses in the experimental (60%) and control (84.6%) groups had work shifts in circulation, and most of the participants were undergraduates (80% in the experimental group and 84.6% in the control group). The chi-square test results revealed no statistically significant difference between the two groups in terms of demographic variables (*p*>0.05) (Table 1).

**Table 1:** Participants’ demographic information in experimental and control groups

Variables		**Experimental group (*n*=25)**	**Control group (*n*=26)**	***p*-value**
		***n* (%)**	***n* (%)**	
Gender	Female	19 (76.0)	17 (65.4)	0.41
male	6 (24.0)	9 (34.6)
Age (years)	<30	19 (76.0)	18 (69.2)	0.43
	31-40	5 (20.0)	8 (30.8)	
	41-50	1 (4.0)	0 (0)	
Marital status	single	12 (48.0)	10 (38.5)	0.49
	married	13 (52.0)	16 (61.5)	
Work experience	<10	23 (92.0)	24 (92.3)	0.99
	11-20	2 (8.0)	2 (7.7)	
Work shift	Morning	5 (20.0)	2 (7.7)	0.15
	Evening	5 (20.0)	2 (7.7)	
	Night	0 (0)	2 (7.7)	
	In circulation	15 (60.0)	20 (76.9)	
Level of education	Bachelor’s	20 (80.0)	22 (84.6)	0.73
	Master’s	5 (20.0)	4 (15.4)	
Type of employment	employed	11 (44.0)	12 (46.2)	0.60
	contractual	5 (20.0)	2 (7.7)	
	Agreement-based	1 (4.0)	2 (7.7)	
Project-based	8 (32.0)	10 (38.5)

Regarding the comparison of the mean score of the nurses’ awareness, attitude, and performance in preventing NI between the experimental and control groups before (T1), immediately (T2), and two months after the intervention (T3), the *t*-test findings indicated no statistically significant difference prior to the intervention (*p*>0.05); however, the differences between the two groups was statistically significant immediately after and two months after the intervention (*p*<0.05) (Table 2). So that immediately and two months after the intervention, the mean score of knowledge, attitude and performance of nurses were significantly higher in the intervention group than in the control group. Also, the results of the analysis of variance with repeated measurements showed that in the experimental group, the changes in the mean score of knowledge, attitude and performance of nurses before, immediately and two months after the intervention were significant, but in the control group, only the changes in the mean score of performance were significant and the changes in the mean score of their awareness and attitude have not been significant (Table 2). The paired comparison of mean score according to the study times in the experimental group showed that the changes in the mean score of knowledge, attitude and performance of nurses increased significantly only immediately after the intervention compared to before the intervention (*p*<0.001) (Table 3). Also, the paired comparison of mean score according to the study times in the control group also showed that the changes in the mean score performance of nurses two months after the intervention compared to before (*p*=0.001) and immediately (*p*=0.001) after the intervention were significantly reduced (Table 4).

**Table 2:** Comparison of mean scores of awareness, attitudes, and performance in preventing NI between experimental and control groups before (T1), immediately (T2), and two months after the intervention (T3)

Variable	Groups	T1		T2		T3		***p*-value**
		Mean	SD	Mean	SD	Mean n	SD	
Awareness	Experimental	7.44	2.06	10.52	2.90	9.28	2.79	0.001
	Control	7.50	1.90	6.77	3.29	6.96	3.22	0.47
***p*-value**		0.94		0.001		0.009		
Attitude	Experimental	35.92	4.68	38.64	2.20	37.56	4.19	0.047
	Control	35.77	3.60	34.19	3.31	34.62	3.84	0.20
***p*-value**		0.90		<0.001		0.006		
Performance	Experimental	57.48	4.84	61.28	12.80	59.00	3.50	0.009
	Control	56.31	8.29	53.27	11.36	43.88	4.45	0.001
***p*-value**		0.51		0.012		<0.001		

**Table 3:** Comparison of changes in the mean score of knowledge, attitude and performance in the experimental group before (T1), immediately (T2) and two months after the intervention (T3)

Variable	Time		Mean Difference	**Adj. *p*-value**
Knowledge	T1	T2	3.08	0.001
T3	1.84	0.27
T2	T3	-1.24	0.10
Attitude	T1	T2	2.72	0.011
		T3	1.64	0.21
	T2	T3	-1.08	0.19
Performance	T1	T2	3.80	0.014
		T3	1.52	0.99
T2	T3	-2.3	0.07

**Table 4:** Comparison of changes in the mean score of performance in the control group before (T1), immediately (T2) and two months after the intervention (T3)

Variable	Time		Mean Difference	Adj. P value
Performance	T1	T2	-3.04	0.44
T3	-12.42	0.001
T2	T3	-9.38	0.001

## Discussion

The present study aimed to examine the effect of the HBM-based educational program on nurses’ awareness, attitudes, and performance in preventing NI in the Imam Reza Hospital in Larestan in 2022. The data analysis results that the nurses’ awareness increased significantly immediately after the intervention compared to the pre-intervention phase; however, the increase was not significant two months after the intervention compared to the pre-intervention phase. In this regard, the HBM constructs, especially perceived threats and perceived severity, seemed to arouse sensitivity to NI among nurses. This HBM-based educational program, especially the two HBM constructs of the perceived threat and perceived severity, aimed at enhancing nurses’ sensitivity and understanding of the risk of exposure to NI and their critical role in preventing the transmission of NI to patients, patient companions, nurses' families, and even the community. Moreover, these two constructs aimed at arousing the feeling of need among nurses to increase their awareness about NI prevention. 

These results are in line with those proposed by other researchers, including Beikmoradi *et al.*,^27^ Jeihouni *et al.*,^28^ Zeigheimat *et al.,*^25^ Darzi Poor *et al*.,^29^ Amiri Siavashani *et al.,*^30^ and Srithongklang *et al.,*^31^ For example, Zeigheimat *et al.* ^(^^25^ concluded that the HBM-based educational intervention improved the nurses’ knowledge about controlling NI, especially about hand washing and injection. Jeihouni *et al*.^28^ also indicated a significant increase in the experimental group’s awareness immediately after and four months after the HBM-based intervention. 

The present findings also indicated a significant increase in the nurses’ means scores of attitudes immediately after the intervention compared to the pre-intervention phase; however, no significant difference was observed two months after the intervention compared to the pre-intervention phase. To conclude, this HBM-based educational program, by addressing the three HBM constructs of perceived benefits, perceived barriers, and self-efficacy, has been effective in promoting the nurses’ attitudes. This is because this program discussed the benefits of proper behavior and measures in preventing NI as well as the barriers and costs imposed by NI. Moreover, there was an exchange of views with the nurses regarding appropriate alternative solutions to remove, modify, or control the NI barriers, one of the main achievements of which was promoting the nurses’ attitude towards their key role in preventing NI and taking appropriate measures to prevent the NI complications and huge costs. This construct cannot be achieved; unless the nurses promote their attitudes and self-confidence by reinforcing their self-efficacy. In this regard, the findings are consistent with those reported by Abdul Rasul *et al.,*^32^ Kim *et* al.,^33^ and Al Elkaradawy *et al*.^34^ For example, Abdul Rasul *et al.*^32^ confirmed the positive effect of HBM-based intervention on the healthcare workers’ attitudes toward NI at the National Liver Institute in Egypt.

The findings also revealed a significant increase in the nurses’ means scores of performances immediately after the intervention compared to the pre-intervention phase; however, no significant difference was observed two months after the intervention compared to the pre-intervention phase. In the control group, the mean score of performance significantly decreased immediately after and two months after the intervention compared to the pre-intervention phase. Furthermore, implementing the HBM-based educational program by the last HBM structure, i.e., guidelines for action, could help nurses in providing an appropriate framework on how to prevent NI. In this study, the previous five constructs, i.e., perceived susceptibility, perceived severity, perceived barriers, perceived benefits, and self-efficacy, in the training sessions had provided the grounds for changing behaviors towards the final goal of the HBM-based intervention, i.e., improving performance in preventing NI, which was facilitated by the last HBM construct. These findings confirm the findings of Zeigheimat *et al.,*^25^ Darzi poor *et al.,*^29^ Kouhi *et al*.,^35^ and Jihouni *et al*.^28^ For example, Zeigheimat *et al*.^25^ showed that the HBM-based educational intervention improved the nurses’ performance in controlling NI. Amiri Siavashani *et al*.^30^ also confirmed that HBM-based training could improve dental students’ performance in terms of infection control.

The findings proposed by Kim *et al*.^33^, Kakkar *et al*.^18^, and Xiong *et al*.^36^ were in contrast with the present findings. However, it should be noted that in Kim et al.’s study, the educational intervention was not based on HBM. In their study, training was done in a compressed manner. Therefore, one of the reasons can be short training course. Also, increasing awareness alone cannot lead to behavior change, and therefore, the use of the health belief model and its structures, especially the structure of perceived barriers, can be effective in removing more barriers and inhibiting factors, and finally, improve performance.To sum up, the present findings documented the effectiveness of the HBM-based educational programs in promoting the nurses’ awareness, attitude, and performance in preventing NI, and these findings were also supported by many studies on awareness, attitudes, and performance. One of the strengths of the current study is that the HBM, which is the most specific educational model in the field of health behavior change, was used for the educational intervention. Also, unlike other studies, the impact of the HBM on the three variables of awareness, attitude, and performance has been studied simultaneously. In this study, the potential and power of the HBM has been used with the aim of changing the health behavior of preventing NI in nurses, while in most of the previous studies, it has been used to change the behavior of patients suffering from acute and chronic disorders. 

Despite the wide range of topics and content of NI training, due to the interference of training sessions with the professional activities of nurses, there were limitations in the number of training sessions used in the implementation of the HBM, which were considered in in the design of the implementation of the model by the research team. 

Conclusion.This study revealed the necessity of HBM-based interventions in promoting nurses’ awareness, attitudes, and performance in preventing NI. The findings also indicate that the implementation of the HBM-based educational program could contribute to changing the nurses’ behaviors with the support of their improved awareness and attitudes by teaching the benefits of compliance with infection control standards and the life, professional, social and economic risks and complications of NI, discussing how to adjust and remove the barriers to NI prevention, and considering the unique role of self-efficacy. It is suggested to investigate the effect of the HBM-based educational program on the knowledge, attitude and performance of nursing students in preventing NI. Also, the effect of other educational models (PRECEDE- PROCEED, BASNEF) on the prevention of NI should also be investigated.

## References

[1] 1. Allegranzi B, Nejad SB, Combescure C, Graafmans W,Attar H, Donaldson L, et al. Burden of endemic healthcare-associated infection in developing countries: systematic review and meta-analysis. Lancet. 2011; 377(9761):228-41. 10.1016/S0140-6736(10)61458-4 21146207

[2] 2. Ducel G, Fabry J, Nicolle L. Guide pratique pour la lutte contre l’infection hospitalière [Internet]; Geneve: WHO; 2008. Available from: https://apps.who.int/iris/handle/10665/69751

[3] 3. Habibzadeh Sh. Knowledge, Attitude, and Practice of ICU Nurses about Nosocomial Infections Control in Teaching Hospitals of Tabriz. Iran J. Nurs. 2010; 23(64):17-28.

[4] 4. Aghakhani N, Sharif Nia H, Ghana S, EmamiZeydi A, Siyadat Panah A, Rahbar N, et al. Surveying Prevention of nosocomial infections among nurses in educational hospitals of Uremia in 2009. Fam. Health. 2012; 1(3): 21-5.

[5] 5. Cardo D, Dennehy PH, Halverson P, Fishman N, Kohn M, Murphy CL, et al. Moving toward elimination of healthcare-associated infections: a call to action. Infect. Control Hosp. Epidemiol. 2010; 31(11):1101-5. 10.1086/656912 20929300

[6] 6. Suchitra J, Devi L. Impact of education on knowledge, attitudes and practices among various categories of health care workers on nosocomial infections. Indian J. Med. Microbiol. 2007; 25(3):181. 10.4103/0255-0857.34757 17901633

[7] 7. Hinkle j, Cheever KH, Oberbaugh K. Brunner & Suddarth's textbook of medical-surgical nursing: Lippincott Williams &Wilkins; 2010.

[8] 8. Cottrell RR, Girvan JT, McKenzie JF, Seabert DM, Stearns PN. (1972). Principles and foundations of health promotion and education (8th ed.). Jones & Barlett Learning; 2023.

[9] 9. Allah-Bakhshian A, Moghaddasian S, Zamanzadeh V, Parvan K, Allah-Bakhshian M. Knowledge, attitude,and practice of ICU nurses about nosocomial infections control in teaching hospitals of Tabriz. Iran J. Nurs . 2010; 23(64):17-28.

[10] 10. Wallace R, Doebbelingn. Public Health and Preventive Medicine. 17^th^ Ed. Stanford; 2009. P273.

[11] 11. Arinze-Onyia S, Ndu A, Aguwa E, Modebe I, Nwamoh U. Knowledge and practice of standard precautions by health-care workers in a tertiary health institution in Enugu, Nigeria. Niger. J. Clin. Pract. 2018; 21(2):149-55. 10.4103/njcp.njcp_69_17 29465047

[12] 12. Kalantarzadeh M, Mohammadnejad E, Ehsani SR, Tamizi Z. Knowledge and practice of nurses about the control and prevention of nosocomial infections in emergency departments. Archi. Clin. Infecti. Dis. 2014; 9(4):182-78.

[13] 13. Yaghubi M, Sharifi S, Abbaspour H. Knowledge, attitude, and practice of intensive care units nurses about nosocomial Infections control in hospitals of Bojnurd in 2012. J. North Khorasan Univ. Med. Sci. 2014; 5(5):943-50.

[14] 14. World Health Organization. Guidelines on hand hygiene in health care first global. Geneva: WHO; 2009. Available from: https://www.who.int/publications/i/item/9789241597906 23805438

[15] 15. Farokhifar M, Gafarian Shirazi H R, Yazdanpanah S. Survey of Knowledge, Attitude and Practice of Nursing Staff About Nosocomial Infection Control in Bushehr. J. Arak Univ. Med. Sci. 2001; 4(4):42-8.

[16] 16. Nasiri A, Balouchi A, Rezaie-Keikhaie K, Bouya S, Sheyback MAL, Rawajfah O. Knowledge, attitude, practice, and clinical recommendation toward infection control and prevention standards among nurses: A systematic review. Am. J. Infect. Control. 2019; 47(7):827-33. 10.1016/j.ajic.2018.11.022 30612817

[17] 17. Hosseini M S, Mazidi Sharafabadi F, Eslami H, Jalili M, Afkhami Aghda M. The Effect of Education on Knowledge, Attitude, and Practice of Hospital Personnel in Preventing Hospital Infections. J. Toloo e Behdasht. 2019; 18(2):70-80.

[18] 18. Kakkar SK, Bala M, Arora V. Educating nursing staff regarding infection control practices and assessing its impact on the incidence of hospital-acquired infections. J. Educ. Health Promot. 2021; 10:40. 10.4103/jehp.jehp_542_20 PMC7933683 33688549

[19] 19. Coppola A, Sasso L, Bagnasco A, Giustina A, Gazzaruso C. The role of patient education in the prevention and management of type 2 diabetes: an overview. Endocrine. 2015; 22: 65-8. 10.1007/s12020-015-0775-7 26494579

[20] 20. Myers R, Larson E, Cheng B, Schwartz A, Da Silva K, Kunzel C. Hand hygiene among general practice dentists: a survey of knowledge, attitudes and practices. J. Am. Dent. Assoc. 2008; 139(7):948-57. 10.14219/jada.archive.2008.0282 18594081

[21] 21. Najafi Ghezeljeh T, Abbasnejad Z, Rafii F, Haghani H. Nurses’ Knowledge, Beliefs and Practices towards Hand Hygiene. Hayat. 2015; 21(1):79-93. (Persian) 10.1016/j.ajic.2015.03.018 25997879

[22] 22. Glanz K, Rimer BK, Viswanath K. Health behavior and health education: theory, research, and practice. 4^th^ ed. John Wiley & Sons; 2008

[23] 23. Namdar AS.Bigizadeh, M.M. Naghizadeh, Measuring Health Belief Model components in adopting preventive behaviors of cervical cancer. J. Fasa Univ. Med. Sci. 2012; 2(1): 34-44.

[24] 24. Masoudy G, KhasheiVarnamkhasti F, Ansarimogadam A, Sahnavazi M, Bazi M. Predication of Compliance to Standard Precautions among Nurses in Educational Hospitals in Zahedan Based on Health Belief Model. Iran J. Health Educ. Health Promot. 2016; 4(1):74-81.

[25] 25. Zeigheimat F, Ebadi A, Rahmati-Najarkolaei F, Ghadamgahi F. An investigation into the effect of health belief model-based education on healthcare behaviors of nursing staff in controlling nosocomial infections. J. Educ. Health Promot . 2016; 5:23. 10.4103/2277-9531.184549 PMC4960766 27500176

[26] 26. Soleimani Z, Mosadeghrad A M, Abbasabadi-Arab M, Moradi M, Abediinjad P, Mesdaghinia A. Designing and Psychometric Testing of an Instrument to Assess the Knowledge, Attitude and Practice of Clinical Staff about Nosocomial Infections. J. Mazandaran Univ. Med. Sci. 2021; 31(197):111-122.

[27] 27. Bikmoradi A, Mardani D, Soltanian A, Khatiban M. The impact of educational evidence-based handwashing program on knowledge, attitude, and adherence of intensive care units’ nurses. Sci. J. Hamadan Nurs. Midwifery Fac. 2013; 21(3):5-13. (Persian).

[28] 28. Jeihooni AK, Kashfi SH, Bah mandost M, Harsini PA. Promoting Preventive Behaviors of Nosocomial Infections in Nurses: The Effect of an Educational program based on Health Belief Model. In vest. Educ. Enferm. 2018; 36(1):e09. 10.17533/udea.iee.v36n1e09 29898348

[29] 29. Darzi Poor M, Tavakoli R, Shojae Zade D, Rezagholizadeh Omran Z. The Effect of an Educational Program on Health Belief Model of Preventive Behaviors of Nosocomial Infection by Babol Hospitals Midwives. J. Arak Univ. Med. Sci . 2022; 25(1): 40-53.

[30] 30. Amiri Siavashani M, Shojaeizadeh D, Azam K. A Study on the Effect of Educational Intervention Based on Health Belief Model on Infection Control Among Dental Students of Shahid Beheshti University of Medical Sciences. J. School. Public. Health Health Res. 2018; 16(1):75-86.

[31] 31. Srithongklang W, Panithanang B, Kompor P, Pengsaa P, Kaewpitoon N, Wakkhuwatapong P, et al. Effect of educational intervention based on the health belief model and self-efficacy in promoting preventive behaviors in a cholangiocarcinoma screening group. J. Cancer Educ. 2019; 34(6):1173-80. 10.1007/s13187-018-1424-7 30244403

[32] 32. Abdel-Rasoul GM, Al Bahnasy RA, Mohamed OA, Abdel-Aziz AM, Mourad WS, Youssef MF. Effect of an educational health program on the knowledge, attitudes and practices of healthcare workers with respect to nosocomial infections in the National Liver Institute, Egypt. Menoufia Med. J. 2016; 29(4):984.

[33] 33. Kim Y, Kim MY, Seo YH. The effects of an intensive education program on hospital infection control on nursing students' knowledge, attitude, and confidence in infection control. J. Korean Biol. Nurs. Sci. 2016; 18(4):318-26.

[34] 34. Elkaradawy SA, Helaly GF, Wahab MM. Effect of an infection control educational programme on anaesthetists’ attitude and anaesthetic field bacterial contamination. Egypt. J. Anaesth. 2012 Apr 1;28(2):149-56.

[35] 35. Kouhi R, Panahi R, Ramezankhani A, Amin Sobhani M, Khodakarim S, Amjadian M. The Effect of Education Based on the Health Belief Model on Hand Hygiene Behavior in the Staff of Tehran Dentistry Centers: A Quasi-Experimental Intervention Study. Health Sci. Rep. 2023. 6;6(7):e1408. 10.1002/hsr2.1408 PMC10324357 37425231

[36] 36. Xiong P, Zhang J, Wang X, Wu TL, Hall BJ. Effects of a mixed media education intervention program on increasing knowledge, attitude, and compliance with standard precautions among nursing students: A randomized controlled trial. Am. J. Infect. Control . 2017; 45(4):389-95. 10.1016/j.ajic.2016.11.006 PMC7115275 27986296

